# Treatment outcomes of using inhalation sedation for comprehensive dental care

**DOI:** 10.1007/s40368-017-0318-4

**Published:** 2018-01-11

**Authors:** M. Madouh, A. BaniHani, J. F. Tahmassebi

**Affiliations:** Leeds School of Dentistry, University of Leeds, Leeds, UK

**Keywords:** Inhalation sedation, Treatment outcomes, Children, General analgesia

## Abstract

**Aim:**

To assess the outcomes of dental treatment under inhalation sedation within a UK specialist hospital setting.

**Methods:**

This was a retrospective cohort study of the case notes of patients under 17 years of age who received dental treatment using inhalation sedation at a UK specialist setting during the period 2006–2011. Treatment outcomes were categorised into five groups: (1) treatment completed as planned, (2) modified treatment completed, (3) treatment abandoned in sedation unit and patient referred for treatment under general analgesia (GA), (4) treatment abandoned in sedation unit and patient referred for treatment under local analgesia (LA), (5) child failed to return to complete treatment.

**Results:**

In total, the case notes of 453 patients were evaluated. The mean age of the patients was 10.3 ± 2.9 years. Treatment was completed successfully in 63.6% of the cases, 15.9% were referred for treatment under GA, 11.2% failed to return to complete the treatment, 7.1% received modified treatment completed, and only 2.2% were referred for treatment under LA. Treatment outcomes were significantly associated with patient`s age (*p* = 0.002). The treatment outcome “treatment abandoned and child referred to be treated under GA” had significantly lower mean patient ages than the other outcomes.

**Conclusions:**

The majority of children referred for inhalation sedation, completed their course of treatment. A significantly higher proportion of those in the younger age group required GA to complete their treatment.

## Introduction

Dental fear and anxiety are well-known barriers for seeking dental care particularly among paediatric patients (Welbury et al. 2012). Both conditions are multifactorial and caused by a complex interaction of genetic, constitutional and environmental factors (King et al. 1997). Dental fear and anxiety have a negative impact on the child’s quality of life as well as on the quality of dental treatment received (Newton et al. 2012). In addition, they can limit a patient`s attendance for treatment.

The implementation of appropriate behaviour management techniques including non-pharmacological and pharmacological techniques can significantly facilitate the attendance and delivery of high quality dental care to dentally anxious patients. In addition, it creates a positive attitude towards the dental environment. It was reported that 23 million people with dental fear would be more willing to visit a dentist if a form of sedation was offered (Girdler and Hill 1998).

Non-pharmacological techniques are used by paediatric dentists to potentially create a positive attitude towards the dental environment and dental procedures so that future dental visits become more comfortable and pleasant (Wright et al. 1987; Chadwick 2002). Pharmacological techniques, such as inhalation sedation (IS) and GA, involve the administration of a drug or combination of drugs that act centrally to help in the management of the patient`s anxiety or disruptive behaviour (Heasman 2008).

IS with nitrous oxide tends to be the first choice for child patients who are able to communicate but unable to tolerate dental treatment with LA alone. It is offered to children with mild to moderate anxiety to facilitate treatment that is anticipated to be complex like comprehensive dental treatment that requires several visits, or multiple extractions (Hosey 2002).

The aim of this study was to assess the treatment outcomes of using IS for comprehensive dental care within a UK specialist hospital setting.

## Materials and methods

### Study design and ethical approval

Approval was obtained from the Dental Research Ethics Committee (DREC), the National Research Ethics Service (NRES) and the Leeds Research and Development Directorate (R&D).

The study sample was identified from clinical dental records of paediatric patients who received dental treatment in the sedation unit at Leeds Dental Institute (LDI) from 2006 to 2011. The source of the patients at LDI was from general dental practitioners (GDPs) and community dental services referred directly for dental treatment. The clinical records were reviewed by the main author (M.M). Clinical records were included in the study if the patient was under the age of 17 years and had received dental treatment under IS at least for one visit by specialists in paediatric dentistry or paediatric postgraduate students under supervision of specialist staff at LDI between 2006 and 2011. Dental records of patients who were over 17 years old, and for whom a decision was made to treat them using means other than IS on their initial assessment visit at the sedation unit were excluded from the study.

Data of patient`s age, gender and treatment outcome were recorded on a proforma by a trained data abstractor (M.M).

### Outcomes of the treatment

The outcomes of dental treatment in the current study were categorised into five groups. This outcome was recorded for each patient upon the termination of their course of dental treatment in the sedation unit (Table 1).

**Table 1:** Description of the outcomes of dental treatment carried out using inhalation sedation (IS) in the study

Treatment Outcome	Definition
Treatment completed as planned	Treatment carried out in accordance with the proposed treatment plan that was documented in the patient’s records
Modified treatment completed	Patient received a modified treatment than that originally planned in the patient’s record (e.g. extraction carried out instead of pulpotomy)
Treatment abandoned in sedation unit and patient referred for treatment under GA	Patients referred for dental treatment under GA due to lack of cooperation
Treatment abandoned in sedation unit and patient referred for treatment under LA	Patient for whom the dental treatment did not require IS and were referred for completion of treatment under LA
Child failed to return to complete treatment	Patient failed to attend appointment in the sedation clinic to complete the planned dental treatment as recorded in patient’s record

*GA* general analgesia, *LA* local analgesia

### Analysis of data

Data was entered into SPSS version 19. Probability values of p < 0.05 were considered statistically significant. One-way ANOVA was used to assess the association between patient age and treatment outcome, while Chi square was used to assess the association between patient gender and treatment outcome.

## Results

### Baseline characteristics

**Fig. 1:**
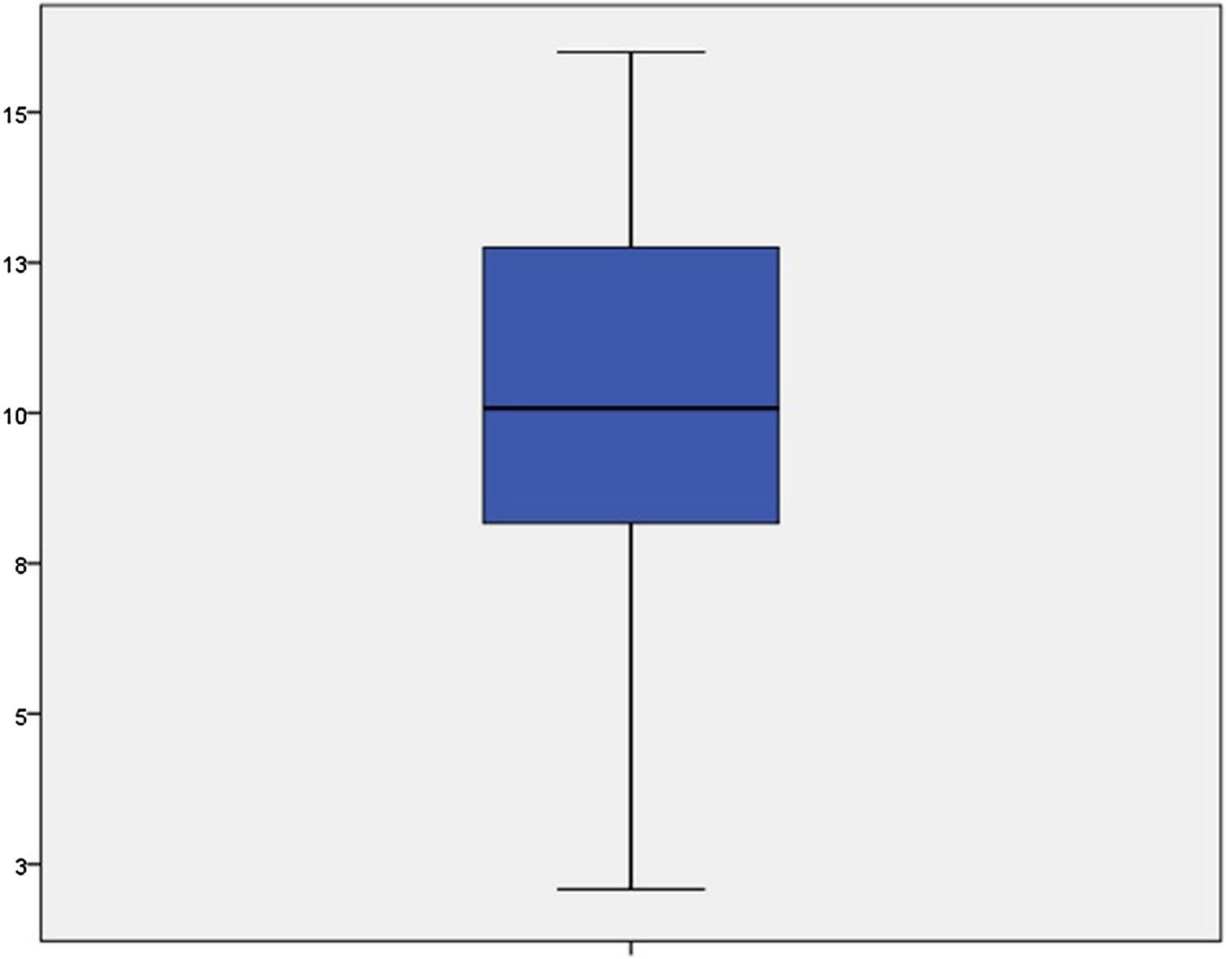
Box plot of the description of patient age in the study

The clinical records of 465 patients were reviewed with 453 case notes fulfilling the inclusion criteria of the study. One patient record was excluded because the patient was over 17 years of age and the remainder were excluded due to the fact that the dental treatment they required at the time of referral was no longer indicated when they attended the sedation unit. There were 240 females (53%) and 213 males (47%). Patients’ age ranged between 2–17 years with the majority in the range of 8.5–13.5 years. The mean age of the patients was 10.30 years ± 2.95 (Fig. 1). Most of the patients in the current study were treated by senior postgraduate paediatric dentistry students.

### Description of treatment outcomes

The treatment outcomes are presented in Table 2. In total 63.3% (n = 288) of the patients completed their dental treatment as planned under IS; 15.9% (n = 72) were referred for completion of dental treatment under GA due to lack of cooperation, 11.2% (n = 51) failed to return for completion of dental treatment, 7.1% (n = 32) received modified dental treatment than that originally planned, and 2.2% (n = 10) were referred for the completion of dental treatment under LA as the proposed dental treatment did not require IS.

**Table 2:** Description of the outcomes of dental treatment carried out under inhalation sedation (IS) in the current study

Treatment outcome	N	%	Mean
Treatment completed	288	63.6	10.4 ± 2.9
Modified & completed	32	7.1	10.8 ± 2.5
Abandoned referred for GA	72	15.9	9.1 ± 2.8
Abandoned referred for LA	10	2.2	11.9 ± 3.1
Failed to attend	51	11.2	10.7 ± 3.3
Total	453	100	10.3 ± 2.9

### The association between patient age and treatment outcome

Patients` age was significantly associated with the outcome ‘Treatment abandoned in the sedation unit and patient referred for treatment under GA’ using one-way ANOVA (*p* = 0.002). The latter outcome had a significantly lower mean patient`s age (9.1 ± 2.8 years) compared with the other outcomes namely treatment completed as planned, modified treatment completed, treatment abandoned in sedation unit and patient referred for treatment under LA, and child failed to return to complete treatment. These outcomes had significantly higher mean patient ages (> 9.1 ± 2.8 years).

### The association between patient gender and treatment outcome

**Fig. 2:**
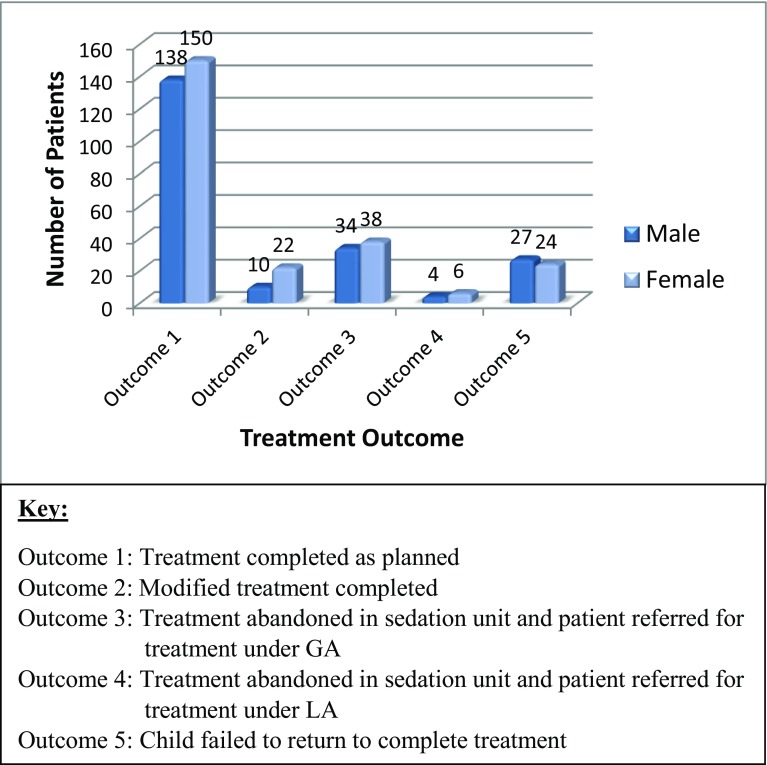
The distribution of treatment outcomes based on patient gender

Figure 2 displays the distribution of treatment outcomes based on patient gender. The proportion of female patients which completed the treatment as planned, received modified dental treatment, and referred for completion of dental treatment under GA or LA was higher than for the number of male patients. Whereas more male compared to female patients in the current study failed to return to complete their dental treatment. These proportions, however, failed to reach a statistical significant difference using Chi square.

## Discussion

This study was a retrospective analysis of the outcomes of dental treatment provided to paediatric patients using IS within a UK specialist hospital setting (LDI).

In the current study, nearly two-thirds of the participants (63.3%) completed their dental treatment as planned under IS. This was a good outcome indicating that children responded well to this form of treatment and that IS is very well accepted among paediatric patients. IS is a viable treatment option instead of GA and for those children who cannot accept dental treatment under LA (Shaw et al. 1996). Also, it indicates that the operators were generally proficient at planning the treatment and providing the IS. The completion of a course of treatment under IS in the present study was noticeably lower than reported previously in the literature (Crawford 1990; Shaw et al. 1996; Bryan 2002). This could be attributed to the differences in the sample size, patient`s age and the nature of the dental treatment provided for patients under IS.

Of the 453 children treated under IS, 15.9% were referred for dental treatment under GA due to lack of cooperation, in accordance with the studies by Crawford (1990) and Shaw et al. (1996). The remaining patients failed to return for completion of dental treatment (11.2%), received modified treatment than that originally planned (7.1%), and only 2.2% were referred for the completion of dental treatment under LA as the proposed dental treatment did not require IS.

The majority of children treated with IS in the current study were between 8.5 and 13.5 years old. This finding was not surprising as younger children are more likely to require GA for the completion of their dental treatment, while older children tend to accept dental treatment carried out using LA only.

The present study`s principal finding was that patient`s age was significantly associated with the outcome of treatment being abandoned in the sedation unit and the child referred to have treatment under GA (*p* = 0.002). It was found that patients who were younger than 10 years old were more likely to require GA for their dental treatment. This finding was in agreement with previous studies which reported that children with mean ages ranging from 3 to slightly above 7 years were more likely to be referred to have their dental treatment under GA (Eidelman et al. 2000; Harrison and Nutting 2000; Camilleri et al. 2004). The lower mean age range seen for GA referral in the previous studies can be explained by the fact that these studies aimed mainly to assess the pattern of referral, disease and treatment for children with ASA-I, II, III, and IV who had received GAs whereas the current study aimed to explore the outcomes of dental treatment using IS in children with ASA-I, and II. Children with ASA-III and IV tend to have comprehensive dental treatment under GA rather than using LA or IS contributing to the lower mean ages seen previously.

No significant association was found between patient’s age and the other treatment outcomes namely treatment completed as planned, modified treatment completed, child failed to return to complete treatment, and treatment abandoned in sedation unit and patient referred for treatment under LA. The latter outcome increased the mean patient ages than the other outcomes (11.9 ± 3.1 years), however, the figures did not reach a significant difference. This indicates that IS is the recommended route for conscious sedation for paediatric dentistry in addition to its numerous advantages (Hosey 2002).

Few studies have explored the influence of gender on treatment outcomes using inhalation sedation (Foley 2005). In disagreement with the literature, the findings of the current study showed no statistical significant association between gender and any of the treatment outcomes. On the other hand, Foley (2005) reported that male patients less than 10 years of age were found to cope better with IS than female patients of the same age. The difference in the findings of both studies can be attributed to the difference in their study designs. Foley carried out a prospective questionnaire-based survey of 50 consecutive patients and their parents with more male than female patients (M: 27; F: 23). While the current study was a retrospective cohort study of 453 case notes with more female than male patients (F: 240; M: 213).

The motive for publishing the current study was to encourage the consideration of providing dental treatment for anxious paediatric patients using IS as a viable option alternative to GA. Although GAs are considered relatively safe in the UK, they are associated with increased risks from the anaesthetic procedure (Harrison and Nutting 2000). Muscle relaxants are required for intubation, and the duration of anaesthesia is prolonged as may be the recovery time. In addition, many children find GAs emotionally traumatic and the experience gained rarely does anything to enable the child to cope with future dental treatment. Indeed the negative impression left by the experience may leave a child in a position where they may be even less amenable to dental care (Harrison and Nutting 2000).

## Conclusions

The majority of the participants (63.3%) in the current study completed their dental treatment as planned under inhalation sedation. Children under 10 years of age were more likely to require GA to facilitate their treatment.
